# Poly(vinyl alcohol)-Controlled Spreading and Film Formation of Poly(3-hexylthiophene-2,5-diyl) at Liquid Interfaces: Influence of PVA Molecular Weight, Degree of Hydrolysis, and Concentration

**DOI:** 10.3390/polym18131674

**Published:** 2026-07-07

**Authors:** Ziyan Shi, Haibin Wang, Huibin Sun, Wei Huang

**Affiliations:** School of Flexible Electronics (Future Technologies), Nanjing Tech University, Nanjing 211816, China; 202361122069@njtech.edu.cn (Z.S.); 202461222230@njtech.edu.cn (H.W.); iamwhuang@njtech.edu.cn (W.H.)

**Keywords:** poly(3-hexylthiophene-2,5-diyl), poly(vinyl alcohol), free spreading, film formation, surface tension

## Abstract

The spreading and film formation of organic polymer solutions on liquid surfaces are key processes in coating, printing, and interfacial processing. However, the mechanisms by which aqueous polymers regulate spreading kinetics and film morphology are not yet fully understood. In this study, the free spreading of Poly(3-hexylthiophene-2,5-diyl) (P3HT)/chlorobenzene solution on poly(vinyl alcohol) (PVA) aqueous surface was employed as a model system to investigate how PVA concentration, molecular weight, degree of hydrolysis, and temperature collectively govern spreading behavior and film formation. Video recording was used to monitor the evolution of the spreading and front-edge morphology, while step-profilometry, UV–visible absorption spectroscopy, and atomic force microscopy were employed to characterize the resulting films in terms of thickness distribution, optical uniformity, and surface roughness. The results reveal that PVA can significantly regulate both the spreading kinetics of P3HT/chlorobenzene droplets and the final film morphology. PVA concentration exhibited a non-monotonic effect on spreading behavior, with intermediate concentrations favoring larger spreading areas and more continuous films. Increasing the PVA molecular weight altered the concentration-dependent spreading window and enhanced asymmetry at the spreading front, whereas reducing the degree of hydrolysis decreased interfacial tension and thereby increased the thermodynamic driving force for spreading, yet the actual spreading rate remained constrained by molecular diffusion, interfacial adsorption, and chain-segment rearrangement. Temperature and a saturated chlorobenzene vapor atmosphere further modulated the interplay among solvent evaporation, interfacial driving force, and viscous dissipation. Under optimized conditions, the resulting P3HT films displayed uniform thickness profiles, consistent optical absorption, and nanoscale surface roughness, and could be repeatedly transferred, assembled into well-defined multilayer structures, and printed onto flexible and curved substrates. These findings demonstrate that PVA aqueous subphase provides a tunable low-shear route for transferable P3HT thin-film fabrication and suggests its potential applicability to other polymer film-forming systems.

## 1. Introduction

Organic optoelectronic polymer thin films serve as key active layers or interfacial functional layers in flexible electronics, photovoltaic devices, organic field-effect transistors, photodetectors, and functional coatings [1,2]. Their thickness, continuity, surface roughness, and lateral uniformity directly affect carrier transport, exciton separation, interfacial contact, and device stability [3,4,5,6]. For most organic optoelectronic devices, the thickness of functional thin films typically ranges from tens to hundreds of nanometers. Therefore, obtaining films with controllable thickness, minimal defects, and uniform morphology over large areas is a critical issue for device integration and scalable fabrication [7,8]. Commonly used solution-processing methods include spin coating, drop casting, blade coating, slot-die coating, and spray coating. Spin coating can readily produce relatively flat films at the laboratory scale, but it suffers from low material utilization and is difficult to adapt to large-area continuous fabrication. Drop casting is strongly affected by solvent evaporation, contact-line pinning, and capillary flow, which can readily lead to coffee-ring formation and thickness nonuniformity. Although blade coating and slot-die coating have potential for scale-up, the resulting film quality is highly dependent on ink rheology, substrate wettability, externally applied shear conditions, and drying-process control [9,10,11]. For the P3HT/chlorobenzene system, the final film morphology is highly sensitive to solvent evaporation, local P3HT concentration gradients, and solute redistribution during drying. On solid substrates, rapid or nonuniform chlorobenzene evaporation can freeze a non-equilibrium polymer distribution, while substrate-dependent wetting and contact-line pinning may further induce thickness variation, edge accumulation, or discontinuous film morphology. Therefore, the development of interfacial processing methods with low shear, low equipment complexity, and suitability for large-area film formation remains an important challenge in the fabrication of organic optoelectronic thin films.

Free spreading and film formation on liquid substrates provide a new strategy for addressing the above challenges. Unlike film-forming methods on solid substrates, in which spreading is driven by external forces, liquid-interface film formation takes advantage of the flexible boundary and low-friction characteristics of the air–liquid interface. This allows polymer solutions to spread spontaneously under the driving force of surface-tension gradients and subsequently form transferable films after solvent evaporation or diffusion [12]. The Langmuir–Blodgett (LB) technique is a representative air–liquid interfacial film-forming method that enables molecular-level assembly and ordered transfer at the liquid surface. However, this method is more suitable for the construction of monolayers or ultrathin films; obtaining thicker films generally requires repeated compression and transfer steps, making the process relatively complex [13,14]. In recent years, spontaneous spreading on water surfaces, the floating-film transfer method, solution-floating approaches, and spatially confined assembly have been used to prepare conjugated polymer and organic semiconductor thin films, demonstrating advantages in terms of simplicity, transferability, and low-shear film formation [15,16,17,18]. However, whether free spreading can proceed effectively depends on the spreading coefficient among the polymer solution, the liquid substrate, and the corresponding interfaces. Only when the spreading driving force is sufficient to overcome interfacial resistance and viscous dissipation can a droplet form a continuously spreading film on the liquid surface [19,20]. Meanwhile, during the rapid evaporation of volatile organic solvents, the solution concentration, local surface tension, and interfacial viscosity continuously change, which can readily lead to insufficient spreading, front rupture, retraction, thickness nonuniformity, or interfacial instability [19,21,22]. The addition of small-molecule surfactants to the aqueous phase can regulate surface tension and promote spreading; however, their rapid adsorption, diffusion, and fluctuations in interfacial concentration may also induce additional Marangoni flows and interfacial disturbances, thereby still limiting the preparation of large-area uniform thin films [17,18].

In view of these challenges, introducing water-soluble polymers with interfacial activity and tunable molecular dynamics into the aqueous phase provides a rational route for regulating free spreading and improving thin-film morphology [23]. In this study, PVA was selected as the polymeric subphase regulator because its surface activity and adjustable interfacial behavior have been well documented [24]. Its molecular weight, degree of hydrolysis, and concentration can be systematically varied, allowing the viscosity, surface properties, and interfacial adsorption behavior of the aqueous subphase to be adjusted in a controlled manner [24,25,26]. Unlike small-molecule surfactants, PVA molecules can undergo slower diffusion, interfacial adsorption, conformational rearrangement, and possible chain entanglement at liquid interfaces, which may simultaneously modify the spreading driving force and flow dissipation [27,28]. Although existing studies on the surface properties, interfacial adsorption behavior, and polymer film preparation of PVA solutions have laid a foundation, there is still a lack of systematic understanding of how aqueous PVA substrates influence the spontaneous spreading of organic polymer solutions at liquid interfaces. More specifically, how the molecular weight, degree of hydrolysis, and concentration of PVA act in concert to control Marangoni flow, viscous dissipation, film continuity and pattern formation remains poorly understood.

The central hypothesis of this work is that PVA does not function merely as a static surface-tension modifier or passive liquid support. Instead, its molecular weight, degree of hydrolysis, and concentration determine a dynamically evolving polymeric interfacial layer, in which polymer diffusion, interfacial adsorption, chain-segment relaxation, interfacial flow resistance, and solvent evaporation are coupled during droplet spreading. This mechanism is distinct from conventional Langmuir–Blodgett assembly, which relies on external compression of interfacial films, and from many floating-film transfer or liquid-interface spreading approaches, in which the liquid subphase is often treated primarily as a supporting medium or a surface-tension-regulating phase. To address this gap, the present study uses the free spreading of a P3HT/chlorobenzene solution on an aqueous PVA substrate as a model system to systematically investigate the effects of PVA concentration, molecular weight, degree of hydrolysis, temperature, and chlorobenzene vapor atmosphere on spreading dynamics and film formation. High-speed imaging was used to quantitatively analyze the evolution of the spreading radius, spreading area, and front morphology, while film thickness, optical absorption, and surface roughness were characterized to establish the relationship among the composition of the aqueous PVA substrate, the interfacial spreading process, and the final film morphology. This study aims to clarify the balance between driving forces and dissipation in PVA-mediated free spreading at the air–liquid interface, thereby providing an experimental basis for the low-shear interfacial processing of transferable organic polymer thin films.

## 2. Materials and Methods

### 2.1. Materials

Three commercial poly(vinyl alcohol) (PVA) grades with different supplier-specified weight-average molecular-weight ranges (Mw) and degrees of hydrolysis (DH) were purchased from Aladdin Biochemical Technology Co., Ltd. (Shanghai, China): Mw = 13,000–23,000 g·mol^−1^, DH = 98%; Mw = 89,000–98,000 g·mol^−1^, DH = 99%; and Mw = 13,000–23,000 g·mol^−1^, DH = 87–89%. These molecular-weight values are the specification ranges provided by the supplier rather than batch-specific exact Mw values. Therefore, the three samples are referred to in this study as low-molecular-weight high-hydrolysis-degree PVA, high-molecular-weight high-hydrolysis-degree PVA, and low-molecular-weight low-hydrolysis-degree PVA, respectively. Tetrabutylammonium bromide (TBAB) was purchased from J&K Scientific Co., Ltd. (Beijing, China). Poly(3-hexylthiophene-2,5-diyl) (P3HT, supplier-specified Mn = 20,000–35,000 g·mol^−1^; dispersity, Đ = Mw/Mn ≈ 3; batch-specific regioregularity not specified) was purchased from Xi’an Yuri Solar Energy. (Xi’an, China). Chlorobenzene was purchased from Sigma-Aldrich (St. Louis, MO, USA). All reagents were used as received without further purification. Ultrapure water with a resistivity of ≥18.2 MΩ·cm was used in all experiments.

### 2.2. Preparation of Aqueous PVA Solutions

Aqueous PVA solutions with concentrations of 0.025, 0.05, 0.075, 0.1, 0.2, 2, 6, and 10 mg/mL were prepared as follows. The required mass of PVA powder was accurately weighed and slowly added to ultrapure water, followed by heating and stirring at 90 °C for 24 h to ensure complete dissolution. After the solution was allowed to cool naturally to room temperature, it was filtered through qualitative filter paper to remove insoluble impurities. Before the spreading experiments, all aqueous PVA solutions were equilibrated at the target temperature for at least 30 min to ensure the thermal stability of the system. After preparation, the PVA aqueous solutions were used in controlled comparative experimental series. In each series, one primary variable, including PVA concentration, molecular weight, degree of hydrolysis, temperature, or chlorobenzene vapor atmosphere, was adjusted while the other experimental conditions were kept constant. This design was used to isolate the main influence of each parameter on interfacial spreading and film formation, rather than to establish a statistically predictive multivariable optimization model. Since spreading in this system is also affected by coupled processes such as solvent evaporation, interfacial transport, and polymer-chain relaxation, selected coupled effects were further examined through comparative experiments, including experiments performed under a chlorobenzene vapor atmosphere.

### 2.3. Preparation of the Polymer Solution for Spreading Experiments

The spreading solution was prepared by dissolving P3HT in chlorobenzene at a concentration of 20 mg/mL. The solution was magnetically stirred until it became homogeneous and transparent and then filtered through a 0.45 μm polytetrafluoroethylene (PTFE) membrane to remove dust and polymer aggregates. Unless otherwise specified, the spreading volume was fixed at 10 μL for all experiments.

### 2.4. Spreading Experiments

The spreading experiments were conducted in a circular polytetrafluoroethylene (PTFE) trough with a characteristic lateral dimension of 12 cm and a liquid depth of 8 mm. Before each experiment, the trough was filled with an aqueous PVA solution. A 10 μL droplet of the organic polymer solution was deposited at the center of the PVA surface using a 2–20 μL adjustable micropipette equipped with a standard disposable pipette tip. During deposition, the micropipette was held vertically, and the tip outlet was positioned approximately 5 mm above the liquid surface. The droplet was released by smoothly depressing the pipette plunger at a low manual speed. The same micropipette, tip type, deposition height, and manual deposition procedure were used for all spreading experiments to minimize variations in droplet initial kinetic energy and initial spreading radius. To reduce possible macroscopic experimental artifacts, the spreading experiments within each comparative series were performed under controlled environmental conditions using the same trough geometry, liquid depth, deposition position, droplet volume, and droplet-deposition procedure. The trough was placed on a horizontal platform, and the PVA aqueous solutions were filtered and thermally equilibrated before use. Each key spreading condition was repeated in independent experiments, and the representative images shown in the figures were selected from reproducible spreading behaviors.

Under uniform illumination, the spreading process was recorded from the top using a Canon EOS 800D camera (Canon Inc., Tokyo, Japan) at 24 frames per second. The recorded videos were used to analyze the macroscopic evolution of spreading area, equivalent spreading radius, and front morphology. Individual frames were extracted at fixed time intervals of 1/24 s and calibrated using the known scale bar. The spreading region was identified from the contrast between the P3HT/chlorobenzene film and the aqueous subphase, followed by image thresholding and manual correction when necessary to avoid errors caused by nonuniform illumination or irregular film edges. The spreading area, A, was calculated from the segmented region, and the equivalent spreading radius was defined as:

(1) $$R_{e}=\sqrt{\frac{A}{\pi }}$$

For strongly asymmetric spreading fronts, the equivalent radius was used as an apparent macroscopic parameter for comparison, while the front morphology was evaluated separately from the time-sequence images. To further describe the apparent spreading kinetics, the apparent radial spreading velocity was calculated from the time-dependent equivalent spreading radius as:

(2) $$v_{r}(t)=\frac{dR_{e}\left(t\right)}{dt}$$

In practice, $v_{r}(t)$ was estimated from the finite difference of $R_{e}\left(t\right)$ between adjacent video frames. Therefore, for asymmetric or petal-like spreading fronts, $v_{r}(t)$ should be regarded as an apparent macroscopic radial velocity rather than a local front velocity.

For temperature-dependent experiments, the trough was placed on a temperature-controlled platform, and the temperature of the PVA solution was controlled within the range of 15–35 °C. For atmosphere-controlled experiments, the PTFE trough was covered with a quartz glass lid containing a circular opening, and a chlorobenzene vapor atmosphere was used to regulate the experimental environment. Pure water and a 10 mg/mL tetrabutylammonium bromide (TBAB) surfactant solution were used as control systems.

### 2.5. Thin-Film Characterization

After the spreading experiments were completed and the organic solvent had fully evaporated, the polymer film formed at the liquid surface was transferred onto a pre-cleaned glass substrate. During transfer, the glass substrate was slowly inserted beneath the liquid surface and then lifted at a steady rate, allowing the polymer film at the liquid surface to adhere to the solid substrate. The transferred samples were dried in a vacuum oven to remove residual surface moisture and were subsequently used for characterization of film thickness, surface morphology, and optical properties.

Film thickness was measured using a stylus profilometer. Before measurement, a well-defined step was created on the film surface, and multiple measurement points were selected from different regions of the sample using a regular hexagonal sampling pattern to obtain the film thickness and its distribution. The average thickness and standard deviation were calculated from the multiple-point thickness data to evaluate thickness uniformity. The optical absorption properties of the films were measured using a UV-1780 ultraviolet–visible spectrophotometer (Shimadzu Corporation, Kyoto, Japan) over a wavelength range of 400–700 nm, with a blank glass substrate used as the reference. To evaluate optical uniformity, absorption spectra were collected from different regions of the same film sample, and differences in absorption-peak shape and intensity among these regions were compared. The surface morphology of the films was characterized by atomic force microscopy (AFM) (Park Systems, Suwon, Republic of Korea) in tapping mode. Different regions of the film were scanned to obtain surface height images, and the corresponding root-mean-square roughness (RMS) values were calculated to analyze surface flatness and local morphological variations.

### 2.6. Surface Tension Testing Calculation Method

In this study, a contact angle goniometer based on the pendant-drop method was used to systematically measure the surface tension of liquids and the interfacial tension between two phases. The measurements included deionized water, chlorobenzene, TBAB aqueous solution, and PVA aqueous solutions with varying molecular weights, degrees of hydrolysis, and concentrations. Additionally, the interfacial tensions of deionized water/chlorobenzene, TBAB aqueous solution/chlorobenzene, and PVA aqueous solution/chlorobenzene systems were also determined. During the experiments, the droplet behavior at the interface was recorded via video, and each frame of the video was analyzed to calculate the corresponding surface and interfacial tensions. The surface tension was computed using the standard pendant-drop method equation:

(3) $$\gamma =\frac{∆\rho gD_{e}^{2}}{H}$$

Here, γ represents the liquid surface tension, ∆ρ is the density difference between the liquid and the gas phase, g is the gravitational acceleration, $D_{e}$ is the equivalent diameter of the droplet, and H is the shape factor. The shape factor H is determined from the droplet’s geometric parameters as follows:

(4) $$\frac{1}{H}=f\left(\frac{D_{s}}{D_{e}}\right)$$

During the pendant-drop measurements, the temporal evolution of surface or interfacial tension was recorded to monitor tension relaxation associated with molecular diffusion and interfacial adsorption. Unless otherwise specified, the values reported in Table 1 and Table S1 correspond to stabilized or near-equilibrium values obtained after the measured tension reached an approximately constant plateau. Therefore, these values were used to estimate the thermodynamic spreading tendency of different systems, rather than to represent the instantaneous surface or interfacial tension at the rapidly moving spreading front. For polymer-containing systems, the time-resolved pendant-drop data were used qualitatively to assess the dynamic interfacial response of the PVA aqueous subphase. However, because the pendant-drop geometry and diffusion pathway differ from those in the free-spreading process, higher-temporal-resolution interfacial measurements would be required to determine the true dynamic tension at the same timescale as the early spreading stage.

## 3. Results and Discussion

### 3.1. Spreading Behavior on Aqueous Subphase

**Figure 1:**
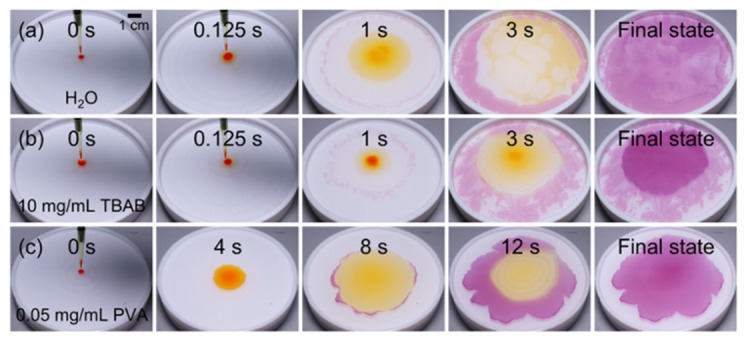
Spreading and film formation process of a 20 mg/mL P3HT/chlorobenzene solution on different aqueous subphases: (**a**) deionized water; (**b**) 10 mg/mL TBAB aqueous solution; and (**c**) 0.05 mg/mL PVA aqueous solution. The PVA has a weight-average molecular weight (Mw) of 13,000–23,000 g·mol^−1^ and a degree of hydrolysis (DH) of 98%. The scale bar is 1 cm.

As shown in Figure 1a, the spreading and film formation behavior of the P3HT/chlorobenzene solution was examined on different liquid subphases, including deionized water, a 10 mg/mL TBAB aqueous solution, and a 0.05 mg/mL PVA aqueous solution (Mw = 13,000–23,000 g·mol^−1^, DH = 98%). The P3HT/chlorobenzene droplets spread spontaneously on all three subphases, but the spreading dynamics and resulting film morphologies differed markedly. On deionized water, the droplet underwent rapid radial expansion immediately after contacting the interface, indicating that the interfacial tension difference between the organic solution and the aqueous subphase was sufficient to drive spontaneous spreading. However, this rapid expansion did not yield a uniform film. As chlorobenzene evaporated and the P3HT concentration increased, pronounced instabilities developed at the spreading front, causing the liquid film to break up into discontinuous droplets. These droplets subsequently spread outward from their own centers, leaving a retained organic-solution region in the center. Additional small droplets were continuously generated at the edge of this retained region and moved toward the periphery until the central droplets were fully dissipated. Consequently, discontinuous, powder-like polymer aggregates remained floating on the water surface.

To improve spreading, small-molecule surfactants are often introduced into the aqueous phase to reduce the interfacial tension between the organic and aqueous phases and thereby increase the spreading coefficient. As shown in Figure 1b, the addition of TBAB markedly altered the spreading behavior of the organic solution. Compared with deionized water, the 10 mg/mL TBAB aqueous solution reduced both the surface tension of the aqueous phase and the interfacial tension between the two phases, with the reduction in interfacial tension being particularly pronounced. This change strengthened the thermodynamic driving force for spreading. Although the P3HT/chlorobenzene droplets still spread rapidly on the TBAB aqueous solution, the resulting film exhibited a darker central region and a nonuniform edge distribution. These observations indicate that increasing the spreading coefficient alone provides only limited improvement in film uniformity and does not eliminate the nonuniform spreading behavior associated with the central retained region.

The spreading coefficient (S) was calculated from the surface tension and interfacial tension according to the following equation:

(5) $$S=\gamma_{1}-\gamma_{2}-\gamma_{12}$$

where $\gamma_{1}$ is the surface tension of the aqueous subphase, $\gamma_{2}$ is the surface tension of the P3HT/chlorobenzene solution, and $\gamma_{12}$ is the interfacial tension between the organic and aqueous phases. As listed in Table 1, the spreading coefficients of deionized water, the 10 mg/mL TBAB aqueous solution, and the 0.05 mg/mL PVA aqueous solution were 1.82, 7.68, and 9.30 mN/m, respectively. All three values are positive, indicating that spontaneous spreading of P3HT/chlorobenzene droplets is thermodynamically favorable on these aqueous subphases. It should be noted that the surface and interfacial tension values used to calculate S were stabilized or near-equilibrium values obtained from pendant-drop measurements, rather than instantaneous dynamic tensions at the rapidly moving spreading front. Therefore, the calculated spreading coefficients should be interpreted as indicators of the initial or near-equilibrium thermodynamic tendency for spreading, rather than as direct measures of the time-dependent spreading coefficient or instantaneous spreading kinetics. During spreading, continuous chlorobenzene evaporation increases the local P3HT concentration within the spreading film, which can dynamically change the surface tension of the organic phase, the organic/aqueous interfacial tension, and consequently the effective spreading coefficient. Thus, the calculated S values are used here only to compare the relative initial or near-equilibrium thermodynamic driving force among different aqueous subphases, whereas the subsequent spreading kinetics and final film morphology are governed by coupled effects of solvent evaporation, solute enrichment, interfacial adsorption, Marangoni flow, and viscous dissipation.

Relative to deionized water, both TBAB and PVA increased the spreading coefficient substantially, with the PVA aqueous solution exhibiting the highest value, suggesting that its static interfacial tension conditions are the most favorable for spreading. Nevertheless, the radius–time curves in Figure S1 show that the actual spreading dynamics do not scale directly with S. On deionized water and the TBAB aqueous solution, the spreading radius increased rapidly and reached the centimeter scale within approximately 1–3 s. Because the videos were recorded at 24 fps, the temporal resolution was sufficient to compare the overall spreading evolution and final spreading extent, but may not fully resolve the earliest rapid acceleration stage, particularly for the deionized-water and TBAB systems. Therefore, the radius–time curves in Figure S1 are interpreted mainly as apparent macroscopic spreading trends rather than as quantitative measurements of sub-second spreading kinetics. By contrast, although the 0.05 mg/mL PVA aqueous solution exhibited the largest spreading coefficient, the spreading radius increased much more slowly at the initial stage before gradually expanding. These results show that the static spreading coefficient reflects only the thermodynamic tendency for spreading and does not by itself determine the spreading rate.

This discrepancy can be understood by considering the spreading process as a dynamic balance between the interfacial driving force and kinetic resistance near the moving front. The stabilized spreading coefficient calculated from the surface and interfacial tension values in Table 1 describes the near-equilibrium thermodynamic tendency for spreading. However, during the actual spreading process, PVA adsorption, chain-segment rearrangement, chlorobenzene evaporation, and P3HT enrichment can continuously modify the surface tension of the aqueous subphase, the surface tension of the organic phase, and the organic/aqueous interfacial tension. At the same time, the viscous resistance of the spreading P3HT/chlorobenzene layer, resistance from the aqueous subphase, and adsorption- or relaxation-related interfacial resistance near the moving front can dissipate part of the driving force. Therefore, the apparent radial growth of the droplet reflects a time-dependent coupling between interfacial thermodynamics and kinetic dissipation, rather than the static spreading coefficient alone.

Based on this interpretation, the combination of a high stabilized spreading coefficient and a slow initial spreading rate in the PVA system indicates that the polymer-containing aqueous subphase introduces a kinetic regulation mechanism distinct from that of small-molecule surfactants. On the one hand, PVA lowers both the surface tension of the aqueous phase and the interfacial tension between the two phases, thereby enhancing the thermodynamic tendency for spreading. On the other hand, the diffusion, adsorption, conformational rearrangement, and segmental relaxation of PVA chains at the air–liquid and liquid–liquid interfaces require finite time and introduce additional resistance to interfacial flow. Possible chain association or entanglement near the interface may further contribute to this kinetic resistance. Chlorobenzene evaporation further increases the local P3HT concentration within the spreading film, which can increase the effective viscosity of the organic phase and shorten the time window available for lateral expansion. As a result, the thermodynamic driving force in the PVA system is partly offset by delayed interfacial response and kinetic dissipation, leading to slower initial spreading and a lower apparent radial spreading velocity than those observed in the deionized-water and TBAB systems.

More importantly, the final film morphologies obtained on the three subphases confirm that a faster spreading rate does not necessarily lead to better film quality. The deionized water system exhibited rapid spreading but produced a fragmented film with poor continuity. The TBAB system, despite its higher spreading coefficient and faster spreading rate, still failed to yield a uniform and continuous film. In contrast, the PVA aqueous solution significantly slowed the spreading process and provided a more effective route for regulating the lateral distribution of the P3HT solution. Taken together, these results show that the role of a surface-active subphase is not limited to increasing the spreading coefficient; rather, its more critical function is to regulate interfacial transport, suppress spreading-front instability, and control solute redistribution during solvent evaporation. This mechanistic distinction provides the basis for the subsequent regulation of PVA-subphase parameters.

### 3.2. Effect of PVA Concentration on the Spreading Process

**Figure 2:**
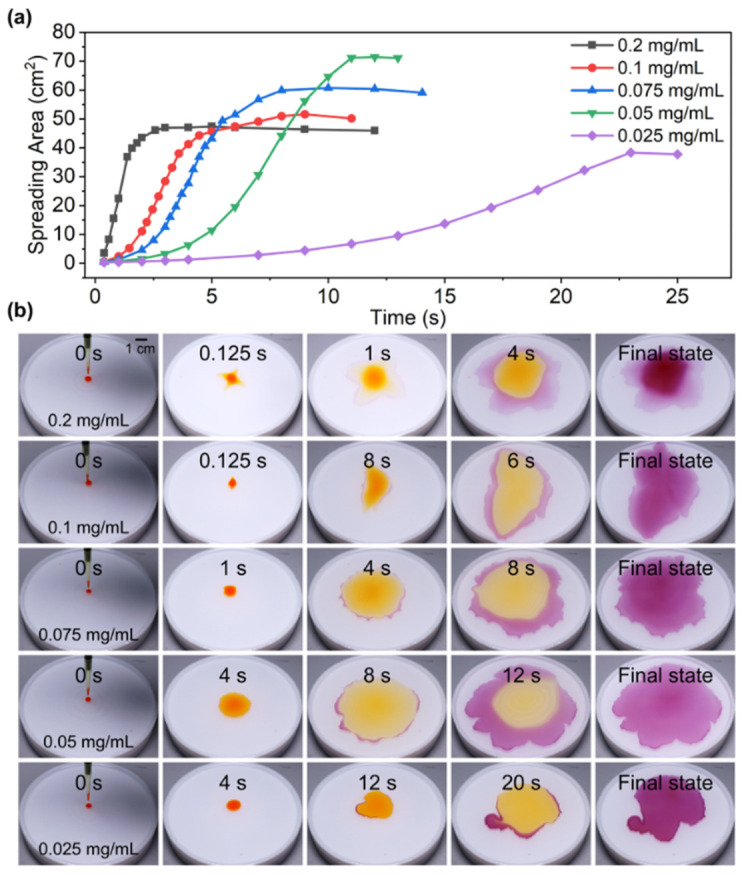
Effect of PVA concentration on the spreading and film formation of a 20 mg/mL P3HT/chlorobenzene solution on PVA aqueous subphases: (**a**) Time-dependent spreading area of the 20 mg/mL P3HT/chlorobenzene solution on PVA aqueous solutions with different concentrations; (**b**) spreading and film formation process of the 20 mg/mL P3HT/chlorobenzene solution on PVA aqueous solutions with varying concentrations. The PVA has a weight-average molecular weight (Mw) of 13,000–23,000 g·mol^−1^ and a degree of hydrolysis (DH) of 98%. The scale bar is 1 cm.

After establishing that the PVA aqueous subphase can significantly alter the spreading behavior of the P3HT/chlorobenzene solution, the effect of PVA concentration on spreading kinetics and the final film morphology was further investigated. In the experiments, the PVA had a fixed weight-average molecular weight (Mw) of 13,000–23,000 g·mol^−1^ and a degree of hydrolysis (DH) of 98%, while the PVA concentration was varied from 0.025 mg/mL to 0.2 mg/mL. As shown in Figure 2a, the spreading area increased with time on all PVA aqueous subphases, but the growth rate, final spreading area, and the time required to reach a steady state differed markedly. Importantly, the dependence of the final spreading area on PVA concentration was clearly nonmonotonic. At relatively high concentrations, namely 0.1 and 0.2 mg/mL, the spreading area increased rapidly at the initial stage and reached a plateau within a short time. However, rapid initial expansion did not translate into improved film uniformity. As shown in Figure 2b, droplets in these high-concentration systems exhibited pronounced irregular expansion during the early stage, and the final films displayed central accumulation morphologies similar to those observed on deionized water and on aqueous subphases containing small-molecule surfactants.

When the PVA concentration was reduced to 0.075 and 0.05 mg/mL, the spreading behavior changed noticeably. Compared with the 0.1 and 0.2 mg/mL systems, spreading at these intermediate concentrations was more sustained, and the spreading area continued to increase over a longer period. Among these samples, the 0.05 mg/mL PVA aqueous solution produced the largest final spreading area, reaching approximately 70 cm^2^, with a more fully expanded spreading front. The corresponding time-sequence images in Figure 2b show that the 0.05 mg/mL system spread relatively slowly during the initial stage, after which the droplet gradually expanded outward to form a large petal-like film. This result suggests that an intermediate PVA concentration provides a more favorable balance between interfacial driving force and interfacial dissipation. At this concentration, PVA molecules are apparently sufficient to modify the interfacial tension and regulate front evolution, yet not so abundant as to impose excessive adsorption-related resistance or near-interface flow resistance. As a result, the spreading front remains mobile for a longer time, allowing sustained lateral expansion of the P3HT/chlorobenzene solution on the aqueous surface. By contrast, when the PVA concentration was further reduced to 0.025 mg/mL, the spreading process slowed markedly, and the final spreading area was smaller than that of the 0.05 mg/mL system. As shown in Figure 2a, the spreading area on the 0.025 mg/mL PVA aqueous solution continued to increase slowly after 20 s, but ultimately reached only approximately 35–40 cm^2^. Figure 2b further shows that at this concentration, the droplet remained confined to a small spreading area for a long time, resulting in an irregular and limited film boundary.

This behavior is likely due to the insufficient number of PVA molecules available at the interface at such a low concentration, limiting effective interfacial adsorption and dynamic regulation of the spreading front, which in turn cannot stabilize the spreading front or sustain the lateral expansion of the P3HT/chlorobenzene droplet. The concentration-dependent viscosity of the PVA aqueous solutions was further examined to clarify the role of bulk solution viscosity in this concentration range. As shown in Figure S2, the relative viscosity of the low-molecular-weight PVA solutions from 0.025 to 0.2 mg/mL remained close to that of deionized water, indicating that bulk viscosity increase and chain entanglement were not dominant factors under these dilute conditions. Therefore, the favorable behavior observed at 0.05 mg/mL is more likely associated with interfacial adsorption and dynamic regulation of the spreading front rather than bulk rheological effects. To further clarify the concentration regime of the PVA aqueous solutions, the overlap concentration, $c^{*}$, was estimated as:

(6) $$c^{*}\approx \frac{1}{\left[\eta \right]}$$

where [η] is the intrinsic viscosity estimated from a reported Mark–Houwink relationship for PVA in water at 30 °C [29]. Based on the supplier-specified molecular-weight ranges, the estimated c* values are approximately 26–36 mg/mL for the low-molecular-weight PVA grades and approximately 11.6–12.3 mg/mL for the high-molecular-weight PVA grade. Therefore, the representative concentration of 0.05 mg/mL is far below the estimated overlap concentration for all PVA grades used in this study, indicating that the PVA chains are in a dilute bulk-solution regime. Accordingly, the interfacial regulation discussed here should not be interpreted as arising from a bulk-overlapped polymer network or a saturated continuous interfacial layer. Instead, the observed spreading regulation is more likely associated with the adsorption and dynamic rearrangement of dilute PVA chains at the air–liquid and liquid–liquid interfaces, together with solvent evaporation and interfacial transport processes.

To further distinguish the effects of PVA concentration from the solvent evaporation process, comparative experiments were conducted in a closed environment. As shown in Figure S3, the spreading time for different PVA concentrations was significantly prolonged under confinement, with the spreading area continuously evolving over tens to hundreds of seconds. Unlike the results obtained under ambient air, the final spreading area in Figure S3 generally increased as the PVA concentration decreased. Notably, the 0.025 mg/mL PVA aqueous solution reached the largest spreading area after prolonged spreading, whereas the final spreading areas for the 0.1 and 0.2 mg/mL systems were relatively smaller. This difference indicates that the effect of PVA concentration on spreading is closely related to the solvent evaporation rate. In an open environment, rapid chlorobenzene evaporation drives quick spreading at the liquid-film front but simultaneously induces solute accumulation and film solidification within a short time, limiting further spreading. In contrast, under a chlorobenzene vapor atmosphere, solvent evaporation is substantially slowed, extending the time during which the droplet remains fluid. In low-concentration PVA systems, the reduced viscous dissipation and weaker interfacial resistance allow the droplet to continue expanding, ultimately achieving a larger spreading area. Conversely, in high-concentration PVA systems, stronger adsorption-related interfacial resistance and near-interface flow resistance still constrain the spreading, resulting in a smaller final area. Therefore, the influence of PVA concentration on film formation is not independent, but is determined by its interplay with solvent evaporation kinetics.

### 3.3. Concentration Dependence and Asymmetric Spreading Behavior in High–Molecular-Weight PVA Systems

**Figure 3:**
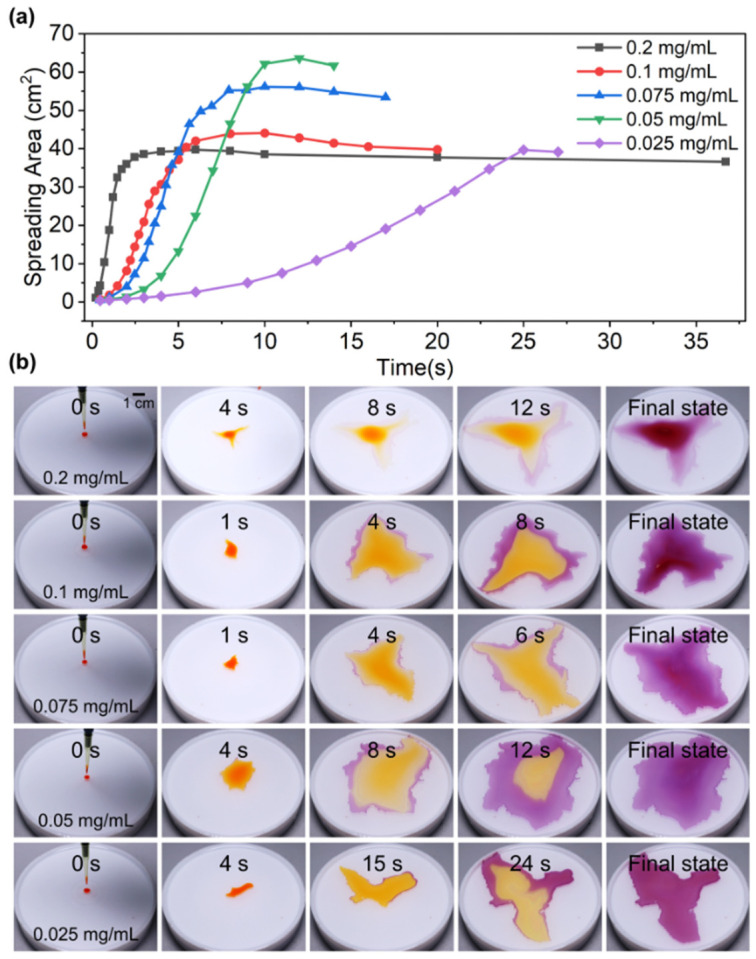
Effect of PVA concentration on the spreading and film formation of a P3HT/chlorobenzene solution on high–molecular-weight PVA aqueous subphases: (**a**) Time-dependent spreading area of a 20 mg/mL P3HT/chlorobenzene solution on high–molecular-weight PVA aqueous solutions with different concentrations; (**b**) spreading and film formation process of the P3HT/chlorobenzene solution on high–molecular-weight PVA aqueous solutions with varying concentrations. The PVA has a weight-average molecular weight (Mw) of 89,000–98,000 g·mol^−1^ and a degree of hydrolysis (DH) of 99%. The scale bar is 1 cm.

Building on the results obtained for the low–molecular-weight PVA systems, the weight-average molecular weight of PVA was further increased to Mw = 89,000–98,000 g·mol^−1^ to investigate how the concentration of high–molecular-weight PVA influences the spreading and film formation of the P3HT/chlorobenzene solution. As shown in Figure 3a, the time-dependent spreading area in the high–molecular-weight PVA aqueous solutions also exhibited a clear concentration dependence; however, the growth rate, maximum spreading area, and final film uniformity varied significantly with concentration. For the 0.2 and 0.1 mg/mL PVA aqueous solutions, the spreading area increased rapidly at the initial stage and reached a plateau within a few seconds, after which only minor changes were observed. This indicates that high-concentration, high–molecular-weight PVA can quickly participate in the interfacial spreading process, but stronger chain-related kinetic resistance and near-interface flow resistance limit further lateral expansion of the droplet, causing the spreading area to stabilize at an early stage.

When the PVA concentration was reduced to 0.075 and 0.05 mg/mL, the spreading process became markedly more prolonged, and the final spreading area increased further. The spreading area on the 0.075 mg/mL PVA aqueous solution reached approximately 55 cm^2^, whereas the 0.05 mg/mL PVA aqueous solution showed more sustained expansion, with the maximum spreading area exceeding approximately 60 cm^2^. Among the concentrations examined in this group, the 0.05 mg/mL system exhibited the largest spreading range. Compared with the high-concentration systems, the 0.05 mg/mL PVA aqueous solution showed a certain delay at the initial stage, followed by a rapid increase in spreading area. This result again points to the existence of an optimal balance between spreading drive and interfacial dissipation. On the one hand, a sufficient number of PVA molecules are still available at the interface to participate in adsorption and regulate the interfacial tension. On the other hand, the interfacial kinetic resistance is not so strong as to completely inhibit lateral expansion of the droplet. Therefore, an intermediate concentration of high–molecular-weight PVA is more favorable for achieving a larger spreading area. By contrast, when the PVA concentration was further reduced to 0.025 mg/mL, the spreading process became significantly slower. Figure 3a shows that the spreading area in this system continued to increase slowly over a relatively long period, and the time required to reach a steady state was markedly longer than that at the other concentrations. However, its final spreading area remained smaller than those of the 0.05 and 0.075 mg/mL systems. This indicates that, at excessively low concentration, interfacial adsorption by PVA is insufficient to rapidly establish an effective interfacial regulation layer, resulting in weak initial spreading and limited front propagation.

Figure 3b further reveals a common morphological feature of the spreading process at different concentrations in the high–molecular-weight PVA systems. Compared with the low–molecular-weight PVA systems discussed above, the P3HT/chlorobenzene droplets on high–molecular-weight PVA aqueous subphases did not exhibit nearly uniform radial expansion; instead, pronounced asymmetric spreading was observed across the entire concentration range. Directional growth of the spreading front occurred to varying degrees in both the high-concentration systems (0.2 and 0.1 mg/mL) and the medium- to low-concentration systems (0.075, 0.05, and 0.025 mg/mL). The final film morphology was often characterized by petal-like structures dominated by two or three principal axes. Even in the 0.05 and 0.075 mg/mL systems, which produced relatively large spreading areas, the spreading front still advanced preferentially along several dominant directions rather than forming a nearly circular film. These reproducible time-sequence observations indicate that increasing the molecular weight of PVA affects not only the extent and rate of spreading, but also the apparent morphological stability of the spreading front.

The directional spreading observed in the high–molecular-weight PVA systems under controlled comparative conditions suggests that the asymmetric morphology is closely related to the molecular-weight-dependent interfacial response of the PVA subphase. This asymmetric spreading behavior is likely associated with the slower diffusion and segmental relaxation dynamics of high–molecular-weight PVA. As the molecular weight increases, the diffusion of polymer chains in the aqueous phase and near the interface becomes slower, prolonging the interfacial response time and allowing more time for adsorption, conformational adjustment, and relaxation of PVA chain segments. Because these interfacial processes may proceed heterogeneously in space, the interface is less likely to maintain a uniform distribution of interfacial stress during spreading. Consequently, local variations in interfacial tension and interfacial flow resistance can develop, giving rise to nonuniform driving forces along the spreading front. Once such anisotropy is established, the droplet tends to expand preferentially along directions of lower resistance or higher local driving force, ultimately producing the observed two- or three-axis petal-like morphology. Therefore, in high–molecular-weight PVA systems, the effect of polymer molecular weight may not be limited to slowing the spreading process, but may also involve the amplification of spatial heterogeneity in interfacial regulation, thereby reducing the apparent symmetry of the spreading front during film formation.

### 3.4. Effect of Degree of Hydrolysis on the Spreading Behavior

**Figure 4:**
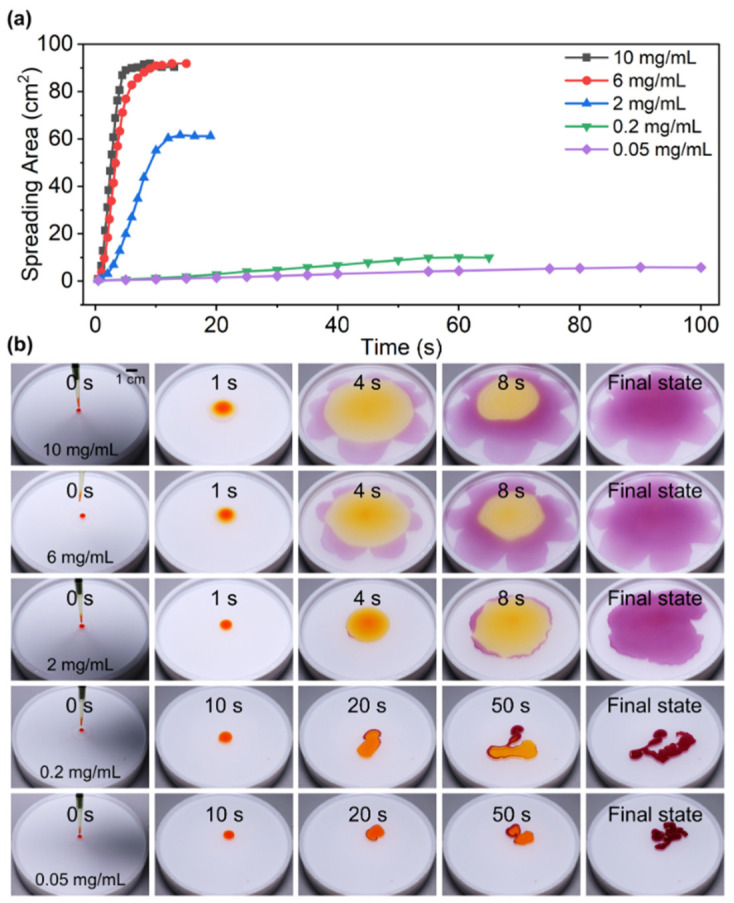
Effect of PVA concentration on the spreading and film formation of a P3HT/chlorobenzene solution on low-hydrolysis-degree PVA aqueous subphases: (**a**) Time-dependent spreading area of a 20 mg/mL P3HT/chlorobenzene solution on low-hydrolysis-degree PVA aqueous solutions with different concentrations; (**b**) spreading and film formation process of the P3HT/chlorobenzene solution on low-hydrolysis-degree PVA aqueous solutions with varying concentrations. The PVA has a weight-average molecular weight (Mw) of 13,000–23,000 g·mol^−1^ and a degree of hydrolysis (DH) of 87–89%. The scale bar is 1 cm.

To further investigate the effect of PVA degree of hydrolysis on interfacial spreading behavior, a low-hydrolysis-degree PVA with Mw = 13,000–23,000 g·mol^−1^ and DH = 87–89% was used as the aqueous subphase and compared with the previously discussed high-hydrolysis-degree PVA system with a similar molecular weight (Mw = 13,000–23,000 g·mol^−1^, DH = 98%). As shown in Figure 4a, the low-hydrolysis-degree PVA system exhibited a pronounced concentration-dependent spreading behavior. At low concentrations of 0.05 and 0.2 mg/mL, the spreading area of the P3HT/chlorobenzene droplets increased slowly and remained relatively small over a prolonged period, indicating that effective lateral expansion was difficult to achieve on low-concentration, low-hydrolysis-degree PVA aqueous subphases. The corresponding time-sequence images in Figure 4b further show that spreading was limited in these systems, and the resulting films were characterized mainly by localized accumulation, discontinuity, and nonuniform morphology. In contrast, as the concentration of low-hydrolysis-degree PVA increased, the spreading behavior improved markedly. When the concentration was increased to 2 mg/mL, the spreading area increased significantly and reached approximately 60 cm^2^ within a relatively short time. When the concentration was further increased to 6 and 10 mg/mL, the spreading area increased rapidly and eventually approached 90 cm^2^. The morphological evolution shown in Figure 4b further indicates that, in the high-concentration low-hydrolysis-degree PVA systems, the droplets covered the aqueous surface more fully, the spreading process became more continuous, and the final film area increased substantially. These results suggest that, for low-hydrolysis-degree PVA, a sufficiently high concentration is required to provide an adequate supply of interfacially active polymer molecules and to establish an effective adsorption layer that can sustain spreading of the P3HT/chlorobenzene solution.

It is noteworthy that the low-hydrolysis-degree PVA system exhibited behavior that was not fully consistent with the static spreading coefficient. According to Table S1, at the same concentration, the low-hydrolysis-degree PVA aqueous solution had a larger spreading coefficient, indicating that, from a thermodynamic perspective, the P3HT/chlorobenzene droplets should have a stronger tendency to spread spontaneously on this subphase. However, Figure 4a,b show that this thermodynamic advantage did not directly translate into faster spreading under low-concentration conditions. Instead, the actual spreading rate remained low, and effective large-area spreading was still difficult to achieve. This observation again indicates that the spreading coefficient reflects only the thermodynamic feasibility of spontaneous spreading and does not directly determine the actual spreading rate or the final film uniformity.

The discrepancy between the larger spreading coefficient and the slower actual spreading suggests that the process in the low-hydrolysis-degree PVA system is strongly constrained by interfacial kinetic factors, which may be further coupled with acetate-group-induced interfacial heterogeneity. Low-hydrolysis-degree PVA contains a higher proportion of residual acetate groups, which gives the polymer chains stronger amphiphilicity and interfacial activity than high-hydrolysis-degree PVA. Previous studies on vinyl acetate–vinyl alcohol copolymers have shown that chain structure can strongly influence rheological relaxation and microstructural behavior [30]. Therefore, the higher acetate-group content may promote local chain association, hydrophobic microdomains, or microphase-separated structures at or near the interface. At the same time, the lower degree of hydrolysis also reduces water affinity and chain hydration, which may affect polymer solubility and hinder chain diffusion in the aqueous phase as well as molecular transport to the newly generated interface. As a result, although the polymer is more surface-active in principle, the establishment of an effective and uniform interfacial regulation layer may still be delayed, particularly at low concentration. Under these conditions, the number of PVA molecules available for interfacial adsorption is limited, and the transfer of polymer chains from the bulk phase to the expanding interface is relatively slow. Consequently, the effective interfacial driving force experienced by the moving front may develop more slowly than the stabilized value estimated from surface and interfacial tension measurements, and the interface cannot be regulated rapidly or uniformly enough to stabilize the spreading front or support sustained lateral expansion. In addition, chlorobenzene evaporation and local P3HT enrichment during spreading may increase the apparent viscosity-related resistance of the organic spreading layer, further limiting radial expansion in the low-concentration regime.

From this perspective, the role of the hydrolysis degree is not simply to alter the static interfacial tension, but to modify the balance between interfacial activity and interfacial supply kinetics. A lower hydrolysis degree increases the amphiphilic character of PVA and can improve its tendency to adsorb at the interface. Nevertheless, if the polymer concentration is too low or if bulk-to-interface transport is too slow, this potential advantage cannot be realized during the short timescale of spreading. In other words, the interface may be thermodynamically favorable for spreading, yet kinetically unable to respond fast enough to maintain uniform front propagation. This interpretation also explains why increasing the concentration of low-hydrolysis-degree PVA markedly improves spreading: a higher polymer concentration enhances the molecular supply to the interface, accelerates the buildup of the adsorption layer, partially compensates for local interfacial heterogeneity, and strengthens the dynamic regulation of the spreading front.

Previous studies have shown that the role of PVA at liquid interfaces is not governed by simple static adsorption, but involves multiple coupled kinetic steps, including bulk diffusion, interfacial adsorption, chain-segment conformational rearrangement, and surface/interfacial tension relaxation [28]. In the present system, these coupled kinetic processes appear to be particularly important for low-hydrolysis-degree PVA. Although this type of PVA exhibits a larger spreading coefficient, the limited molecular supply to the interface and the slower establishment of interfacial regulation at low concentration still suppress the actual spreading rate. Therefore, the effect of hydrolysis degree on spreading should be understood as a coupled consequence of thermodynamic driving force, polymer solubility, acetate-group-induced interfacial heterogeneity, interfacial adsorption kinetics, apparent viscosity-related resistance, and dynamic front stabilization, rather than as a simple change in spreading coefficient alone.

### 3.5. Effect of Temperature on Spreading and Film Formation Behavior

**Figure 5:**
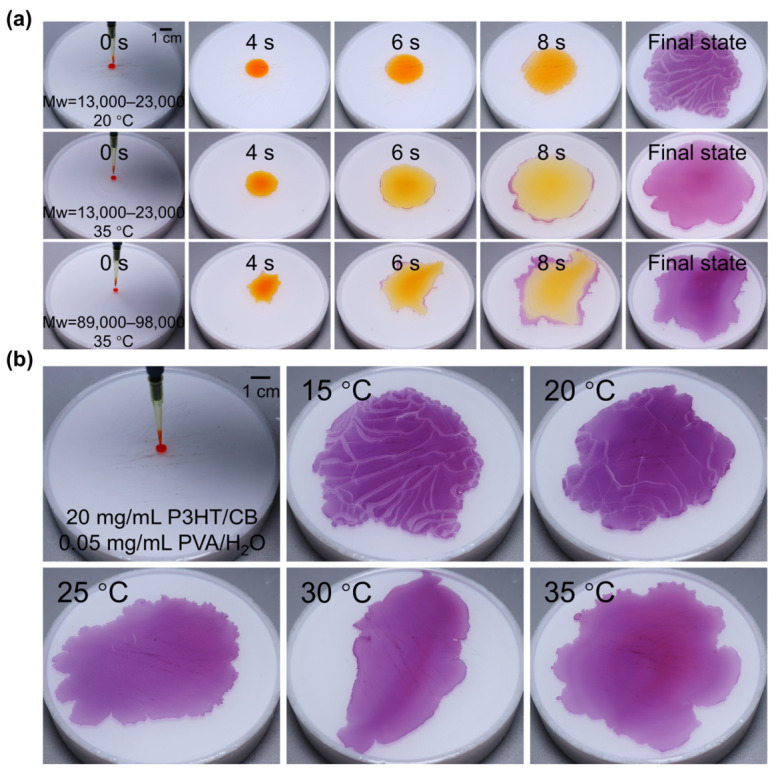
Effect of temperature on the spreading and film formation of a P3HT/chlorobenzene solution on PVA aqueous subphases: (**a**) Spreading and film formation process of a 20 mg/mL P3HT/chlorobenzene solution on PVA aqueous solutions with different temperatures and molecular weights. The conditions are, respectively, a PVA aqueous solution with Mw = 13,000–23,000 g·mol^−1^ and DH = 98% at 20 °C, a PVA aqueous solution with Mw = 13,00023,000 g·mol^−1^ and DH = 98% at 35 °C, and a PVA aqueous solution with Mw = 89,000–98,000 g·mol^−1^ and DH = 99% at 35 °C. Unless otherwise specified, the PVA concentration was 0.05 mg/mL. (**b**) Final film morphologies formed by a 20 mg/mL P3HT/chlorobenzene solution on the surface of a 0.05 mg/mL PVA aqueous solution at different temperatures. The PVA has a weight-average molecular weight (Mw) of 13,000–23,000 g·mol^−1^ and a degree of hydrolysis (DH) of 98%. The scale bar is 1 cm.

In addition to the molecular parameters of PVA, temperature is another important external factor affecting the spreading and film formation of P3HT/chlorobenzene solutions on PVA aqueous subphases. Temperature simultaneously influences molecular diffusion in the P3HT/chlorobenzene phase, the evaporation rate of chlorobenzene, the viscosity of the PVA aqueous subphase, and interfacial molecular rearrangement. Accordingly, a 0.05 mg/mL PVA aqueous solution was used as a fixed-concentration model system for the temperature-dependent experiments. This dilute concentration minimized concentration-induced viscosity and chain-overlap effects, allowing the influence of temperature on spreading behavior to be examined under comparable subphase conditions. As shown in Figure 5a, increasing temperature significantly accelerated the spreading of P3HT/chlorobenzene droplets on the PVA aqueous subphase. At lower temperatures, lateral expansion of the droplets was slow, and the spreading area remained small over a prolonged period, indicating that the spreading process was strongly hindered under low-temperature conditions. This behavior can be attributed to reduced molecular mobility in both the organic phase and the interfacial region, together with the relatively high viscosity of the aqueous subphase. As the temperature increased, the droplets reached a larger spreading area within a shorter time, indicating that elevated temperature facilitates more rapid expansion of the P3HT/chlorobenzene solution at the air–liquid interface. However, Figure 5a also shows that, for high–molecular-weight PVA, noticeable irregularities at the spreading front persisted even at 35 °C, suggesting that increasing temperature can accelerate spreading but cannot completely eliminate the constraints associated with delayed chain relaxation, possible chain association, and near-interface flow resistance.

Figure 5b further illustrates the final film morphologies obtained at different temperatures. At low temperatures, the films exhibited pronounced local inhomogeneity and phase-separation-like features, indicating that the P3HT/chlorobenzene solution did not achieve a sufficiently uniform molecular distribution during spreading and solvent evaporation. It should be emphasized that these morphological features are more directly associated with solute accumulation, diffusion limitation, and solidification within the P3HT/chlorobenzene film than with the intrinsic structure of the PVA aqueous subphase itself. As the temperature increased, the mobility of both P3HT molecules and chlorobenzene increased, allowing more effective molecular rearrangement before film solidification and thereby improving film continuity and overall uniformity. From the perspective of molecular dynamics, elevated temperature enhances diffusion and interfacial flow within the spreading P3HT/chlorobenzene layer, while also promoting chain-segment motion and interfacial tension relaxation near the PVA aqueous interface. As a result, the spreading process becomes more complete and the final film morphology becomes more uniform.

Taken together, these results indicate that the effect of temperature on film formation arises from its coupled influence on molecular mobility, diffusion kinetics, solvent evaporation, and interfacial rearrangement. Moderate heating is beneficial because it simultaneously reduces kinetic limitations on spreading and improves molecular redistribution during film formation. In this sense, temperature acts as a dynamic control parameter that regulates not only the spreading rate, but also the competition among lateral expansion, solvent loss, and structural reorganization within the film. Nevertheless, the beneficial effect of heating is not unlimited. Excessively high temperature may accelerate chlorobenzene evaporation, shorten the time available for interfacial leveling and molecular rearrangement, and intensify local surface-tension gradients, thereby leading to edge undulations or local thickness variations. Therefore, the role of temperature should be understood as a balance between diffusion enhancement and evaporation acceleration, rather than as a simple monotonic promotion of spreading and film uniformity.

### 3.6. Dynamic Interfacial Properties of Different PVA Aqueous Solutions

To further elucidate how the molecular weight and degree of hydrolysis of PVA regulate the spreading and film formation of P3HT/chlorobenzene droplets, 0.05 mg/mL PVA aqueous solutions were selected as representative systems for analyzing their dynamic surface tension, interfacial tension, and actual spreading behavior under a chlorobenzene vapor atmosphere. The selected samples were low–molecular-weight, high-hydrolysis-degree PVA (Mw = 13,000–23,000 g·mol^−1^, DH = 98%), high–molecular-weight, high-hydrolysis-degree PVA (Mw = 89,000–98,000 g·mol^−1^, DH = 99%), and low–molecular-weight, low-hydrolysis-degree PVA (Mw = 13,000–23,000 g·mol^−1^, DH = 87–89%). By combining dynamic interfacial measurements with actual spreading observations, this section aims to clarify how thermodynamic driving force and interfacial kinetics jointly determine spreading behavior.

**Figure 6:**
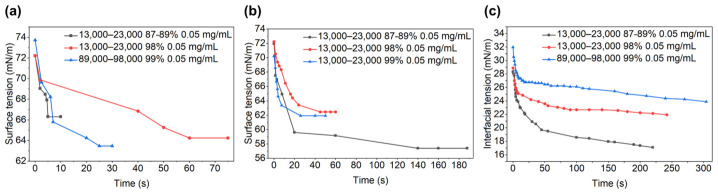
Surface tension and interfacial tension of 0.05 mg/mL PVA aqueous solutions with different molecular weights and degrees of hydrolysis: (**a**) Surface tension of the three PVA aqueous solutions under an air atmosphere; (**b**) surface tension of the three PVA aqueous solutions under a chlorobenzene atmosphere; (**c**) interfacial tension between the three PVA aqueous solutions and chlorobenzene. The PVA samples are low–molecular-weight, low-hydrolysis-degree PVA (Mw = 13,000–23,000 g·mol^−1^, DH = 87–89%), low–molecular-weight, high-hydrolysis-degree PVA (Mw = 13,000–23,000 g·mol^−1^, DH = 98%), and high–molecular-weight, high-hydrolysis-degree PVA (Mw = 89,000–98,000 g·mol^−1^, DH = 99%).

As shown in Figure 6a, under an air atmosphere, the surface tension of all three PVA aqueous solutions decreased with time, reflecting the kinetic processes of PVA diffusion from the bulk phase to the interface, interfacial adsorption, and chain-segment conformational rearrangement. The low-hydrolysis-degree PVA exhibited the highest final surface tension (69.03 mN/m), whereas the low–molecular-weight, high-hydrolysis-degree PVA and high–molecular-weight, high-hydrolysis-degree PVA decreased to 64.24 and 63.47 mN/m, respectively. This result suggests that, at low concentration, the interfacial supply of low-hydrolysis-degree PVA remains insufficient under ambient conditions, thereby limiting its ability to reduce the surface tension of the aqueous subphase. Under a chlorobenzene vapor atmosphere (Figure 6b), the surface tension of all three PVA aqueous solutions decreased further, with the most pronounced reduction observed for the low-hydrolysis-degree PVA, which reached 57.40 mN/m. This stronger response implies that the presence of chlorobenzene vapor facilitates interfacial reorganization of the more amphiphilic low-hydrolysis-degree PVA chains, allowing their hydrophobic segments to orient more effectively at the interface. Figure 6c shows the interfacial tension between the PVA aqueous solutions and chlorobenzene: the low-hydrolysis-degree PVA exhibited the lowest value (17.07 mN/m), whereas the high–molecular-weight, high-hydrolysis-degree PVA showed the highest value (23.87 mN/m). These data indicate that low-hydrolysis-degree PVA has the strongest tendency for interfacial adsorption and can provide the largest thermodynamic driving force for spreading.

The spreading coefficients listed in Table S1 further quantify the thermodynamic driving force of the different PVA aqueous solutions. Under an air atmosphere, the low-hydrolysis-degree PVA exhibited the largest spreading coefficient (18.96 mN/m), whereas the high–molecular-weight, high-hydrolysis-degree PVA showed the smallest value (6.60 mN/m). If spreading were governed solely by static interfacial thermodynamics, the low-hydrolysis-degree PVA system would be expected to exhibit the fastest spreading and the best film uniformity. However, Figure S4 shows that, under a chlorobenzene vapor atmosphere, the actual spreading behavior did not fully follow this expectation. The low-hydrolysis-degree PVA system did not exhibit the highest spreading rate; instead, local accumulation was still observed at the initial stage, and thickness variations remained in the final film. By contrast, the low–molecular-weight, high-hydrolysis-degree PVA system showed a relatively fast spreading rate and formed a comparatively continuous film. Meanwhile, the high–molecular-weight, high-hydrolysis-degree PVA system, despite its comparable thermodynamic tendency for spreading, exhibited a strongly directional and asymmetric spreading front.

Although the time-resolved pendant-drop measurements provide useful information on the relaxation behavior of PVA-containing interfaces, they should not be regarded as direct measurements of the instantaneous surface or interfacial tensions at the expanding spreading front. Taken together, the results in Figure 6 and Figure S4, and Table S1 indicate that the regulation of P3HT/chlorobenzene droplet spreading and film formation by the PVA aqueous subphase cannot be explained by the static spreading coefficient alone. Rather, the actual spreading behavior is determined by the coupling between thermodynamic driving force and dynamic interfacial response, including bulk diffusion, interfacial adsorption, chain-segment rearrangement, and surface-tension relaxation. In the low-hydrolysis-degree PVA system, although the interfacial driving force is relatively large, the interfacial kinetic response remains limited at low concentration, so the potential thermodynamic advantage cannot be converted efficiently into rapid and uniform spreading. In the high–molecular-weight, high-hydrolysis-degree PVA system, slower chain relaxation and stronger spatial heterogeneity in interfacial resistance promote asymmetric spreading. In contrast, the low–molecular-weight, high-hydrolysis-degree PVA system achieves a more favorable balance between spreading drive and kinetic resistance, thereby facilitating continuous droplet expansion and the formation of a relatively uniform film. Therefore, the dynamic interfacial properties of the PVA aqueous subphase are the key factors governing its ability to regulate spreading behavior and final film morphology.

### 3.7. Film Morphology, Uniformity, and Transferability

**Figure 7:**
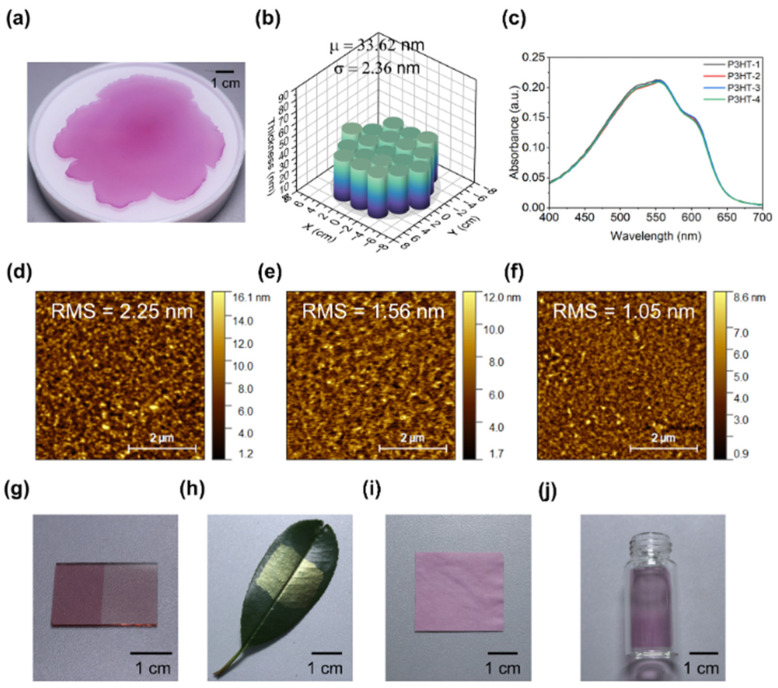
Characterization of the morphology, uniformity, and transferability of P3HT films obtained by spreading on a 0.05 mg/mL PVA aqueous solution. The PVA has a weight-average molecular weight (Mw) of 13,000–23,000 g·mol^−1^ and a degree of hydrolysis (DH) of 98%: (**a**) Optical photograph of the P3HT film formed on the PVA aqueous subphase; (**b**) step-profiler thickness distribution of the transferred P3HT film, with an average thickness of 33.62 nm and a standard deviation of 2.36 nm; (**c**) UV–visible absorption spectra collected from different regions of the same film, where P3HT-1 to P3HT-4 denote different measurement positions; (**d**) AFM height image of the top surface of the film, with an RMS roughness of 2.25 nm; (**e**) AFM height image of the bottom surface of the film, with an RMS roughness of 1.56 nm; (**f**) AFM height image of a spin-coated P3HT film, with an RMS roughness of 1.05 nm; (**g**) stacked P3HT film prepared by repeated transfer; (**h**–**j**) optical photographs of P3HT films transferred onto a leaf, paper, and the curved wall of a glass bottle, respectively. The scale bars in the AFM images are 2 μm, and those in the macroscopic photographs are 1 cm.

After establishing that PVA aqueous subphases can effectively regulate the spreading of P3HT/chlorobenzene solutions, the resulting P3HT films were further characterized in terms of their macroscopic morphology, thickness uniformity, optical uniformity, surface roughness, and transferability. A 0.05 mg/mL PVA aqueous solution was selected as a representative subphase, with Mw = 13,000–23,000 g·mol^−1^ and DH = 98%. As shown in Figure 7a, the P3HT/chlorobenzene solution spread on the PVA aqueous subphase to form large-area, continuous P3HT films, demonstrating that PVA-mediated air–liquid interfacial spreading enables macroscopic film formation under mild conditions. To evaluate lateral thickness uniformity, the transferred P3HT films were measured at multiple locations using a step profiler. As shown in Figure 7b, the thickness values obtained at different positions were narrowly distributed, with an average thickness of 33.62 nm and a standard deviation of 2.36 nm, corresponding to a coefficient of variation of approximately 7.0%, which indicates good thickness uniformity over centimeter-scale regions.

The lateral optical uniformity of the films was further assessed by UV–visible absorption spectroscopy at different positions. As shown in Figure 7c, the absorption spectra collected from four locations (P3HT-1 to P3HT-4) nearly overlapped and displayed the characteristic visible-light absorption features of P3HT films. The minimal differences in peak shape and intensity among these positions indicate that the films possess comparable effective thickness and optical response across the measured area. This observation is consistent with the step-profiler results and further confirms that PVA-mediated air–liquid interfacial spreading can produce laterally uniform P3HT films.

To analyze the microscopic surface morphology, both the top and bottom surfaces of the spread films were characterized by AFM and compared with spin-coated P3HT films. Figure 7d shows that the RMS roughness of the top surface of the spread film was 2.25 nm, whereas Figure 7e shows that the bottom surface had an RMS roughness of 1.56 nm, which is lower than that of the top surface. This difference is likely associated with the distinct interfacial environments experienced during film formation. The top surface is directly exposed to air and volatile chlorobenzene, where solvent evaporation, solute accumulation, and interfacial disturbances can induce local surface undulations. In contrast, the bottom surface is in contact with the PVA aqueous subphase, where the compliant liquid interface and interfacial regulation favor the formation of a smoother contact plane. For comparison, the RMS roughness of spin-coated P3HT films was 1.05 nm (Figure 7f). Although the spread films exhibited slightly higher roughness than the spin-coated films, their roughness still remained within the nanometer range, indicating that air–liquid interfacial spreading can produce relatively flat P3HT films without relying on high-speed spin coating. Therefore, the improved thickness uniformity, surface morphology, and optical uniformity discussed in this section are specific to the P3HT films prepared using the selected 0.05 mg/mL PVA aqueous subphase. A systematic evaluation of film quality across different PVA molecular weights, degrees of hydrolysis, and concentrations will be addressed in future work.

Beyond single-layer film formation, this method also exhibited good repeatable transferability and substrate adaptability. As shown in Figure 7g, repeated spreading and transfer produced stacked P3HT films, demonstrating the feasibility of constructing multilayer structures through this air–liquid interfacial process. Furthermore, the resulting P3HT films could be transferred not only onto conventional planar substrates, but also onto leaves, paper, and curved glass surfaces (Figure 7h–j). Despite differences in surface roughness, flexibility, wettability, and geometry, the films maintained visibly continuous coverage after transfer, indicating good substrate compatibility and morphological adaptability. These results demonstrate that PVA-mediated air–liquid interfacial spreading can produce transferable P3HT films with good thickness uniformity, optical uniformity, multilayer stacking capability, and adaptability to complex substrates, highlighting its potential as a low-shear interfacial film-formation strategy. These transfer results should be regarded as proof-of-concept demonstrations of film transferability rather than a complete evaluation of application-level reliability. For practical use in flexible substrates or device-related systems, further studies will be required to evaluate adhesion stability, bending-cycle durability, repeated transfer yield, multilayer thickness control, and device-relevant electrical or optoelectronic performance.

To further examine whether the PVA-mediated interfacial regulation is limited to the P3HT/chlorobenzene model system, preliminary spreading tests were conducted using several other polymer/chlorobenzene solutions, including F8BT, SY, PFO, and PVK. As shown in Figure S5, compared with the corresponding deionized-water control systems, the PVA aqueous subphase improved the lateral spreading and/or film continuity of these polymer solutions to different extents. These observations suggest that the regulatory effect of PVA is not exclusive to P3HT and may be extendable to other polymer film-forming systems. However, the effectiveness of this strategy is expected to depend on the specific polymer/solvent/subphase combination, including polymer solubility, solvent volatility, solvent–subphase miscibility, surface and interfacial tensions, and the dynamic response of the liquid subphase. Therefore, these results should be regarded as preliminary evidence for broader applicability, while systematic studies involving different solvent systems and liquid subphases are still required.

## 4. Conclusions

The free spreading and film formation of P3HT/chlorobenzene solution on aqueous PVA subphases were systematically investigated. The results demonstrate that the PVA aqueous subphase effectively regulates spreading behavior and enables the formation of large-area, transferable P3HT films at the air-liquid interface. Compared with deionized water and TBAB aqueous solution, the PVA system provided better control over film continuity and uniformity. The results show that the static spreading coefficient mainly reflects the thermodynamic tendency for spontaneous spreading, but it cannot directly determine the actual spreading rate or final film morphology. The concentration-, molecular-weight-, and hydrolysis-degree-dependent experiments suggest that the kinetic limitations observed in this system are closely related to the interfacial supply, adsorption, and dynamic rearrangement of PVA chains near the moving front. These interfacial kinetic processes determine whether the thermodynamic driving force can be effectively converted into sustained and uniform lateral spreading. Temperature and chlorobenzene evaporation further modulate this process by affecting solvent loss, local polymer enrichment, and interfacial transport. An appropriate PVA concentration provided the most favorable balance between spreading drive and kinetic resistance, while increasing molecular weight promoted asymmetric spreading and lowering the degree of hydrolysis enhanced interfacial activity but introduced kinetic limitations at low concentration. Temperature further affected spreading and film formation through its coupled influence on molecular mobility, interfacial rearrangement, and evaporation rate. Under optimized conditions, PVA-mediated air-liquid interfacial spreading produced P3HT films with good thickness uniformity, optical uniformity, smooth surface morphology, repeatable transferability, and adaptability to diverse substrates. These findings demonstrate that aqueous PVA subphases offer an effective low-shear strategy for the fabrication of transferable P3HT thin films and provide useful insight into the dynamic regulation of spreading and film formation at liquid interfaces. Preliminary tests with other polymer/chlorobenzene solutions further suggest that this PVA-mediated regulation may be extended beyond the P3HT/chlorobenzene model system. Nevertheless, its applicability to broader polymer/solvent systems and other liquid subphases should be further evaluated by considering polymer solubility, solvent volatility, solvent–subphase miscibility, surface and interfacial tensions, and dynamic interfacial response.

## Figures and Tables

**Table 1:** Surface tension, interfacial tension, and spreading coefficient of deionized water, TBAB aqueous solution, and PVA aqueous solution systems.

Liquid Subphase	Aqueous Surface Tension (mN/m)	Organic Surface Tension (mN/m)	Interfacial Tension (mN/m)	Spreading Coefficient (mN/m)
Deionized water	72.60	33	37.78	1.82
10 mg/mL TBAB solution	64.75	33	24.07	7.68
0.05 mg/mL PVA solution	64.24	33	21.94	9.30

The PVA has a weight-average molecular weight (Mw) of 13,000–23,000 g·mol^−1^ and a degree of hydrolysis (DH) of 98%. The spreading coefficient was calculated according to $S=\gamma_{1}-\gamma_{2}-\gamma_{12}$.

## Data Availability

The original contributions presented in this study are included in the article/Supplementary Material. Further inquiries can be directed to the corresponding author.

## Supplementary Materials

The following supporting information can be downloaded at: https://www.mdpi.com/article/10.3390/polym18131674/s1, Figure S1: Temporal evolution of the spreading radius of a 20 mg/mL P3HT/chlorobenzene solution on different aqueous subphases; Figure S2: Concentration-dependent relative viscosity of low-molecular-weight PVA aqueous solutions measured using an Ubbelohde viscometer at 25 °C; Figure S3: Time-dependent spreading area of a 20 mg/mL P3HT/chlorobenzene solution on PVA aqueous subphases with different concentrations under a chlorobenzene vapor atmosphere; Figure S4: Spreading and film-formation processes of a 20 mg/mL P3HT/chlorobenzene solution on 0.05 mg/mL PVA aqueous subphases with different molecular weights and degrees of hydrolysis under a chlorobenzene vapor atmosphere; Figure S5: Comparison of spreading and film formation of different polymer/chlorobenzene solutions on deionized water and PVA aqueous solution surfaces; Table S1: Surface tension, interfacial tension, and spreading coefficient of 0.05 mg/mL PVA aqueous solutions with different molecular weights and degrees of hydrolysis.

## Abbreviations

The following abbreviations are used in this manuscript:

PVA	Poly(vinyl alcohol)
P3HT	Poly(3-hexylthiophene-2,5-diyl)
TBAB	Tetrabutylammonium bromide
SY	Super Yellow poly(p-phenylene vinylene)
F8BT	Poly(9,9-dioctylfluorene-alt-benzothiadiazole)
PFO	Poly(9,9-di-n-octylfluorene)
PVK	Poly(N-vinylcarbazole)
PTFE	Polytetrafluoroethylene
AFM	Atomic force microscopy
RMS	Root-mean-square roughness
UV–vis	Ultraviolet–visible spectroscopy
Mw	Weight-average molecular weight
DH	Degree of hydrolysis
