# Supplementary material for: Poly(vinyl alcohol)-Controlled Spreading and Film Formation of Poly(3-hexylthiophene-2,5-diyl) at Liquid Interfaces: Influence of PVA Molecular Weight, Degree of Hydrolysis, and Concentration
Source: Polymers (Basel). 2026 Jul 7;18(13):1674. doi: 10.3390/polym18131674 (PMC13364423; doi:10.3390/polym18131674)
Supplement: Supplementary file 1 [file polymers-18-01674-s001.zip › polymers-4398572-supplementary.pdf]

polymers

Supporting Information

Poly(vinyl alcohol)-Controlled Spreading and Film Formation of Poly(3-hexylthiophene-2,5-diyl) at Liquid Interfaces: Influence of PVA Molecular Weight, Degree of Hydrolysis, and Concentration

Ziyan Shi[1], Haibin Wang[1], Huibin Sun[1,*] and Wei Huang[1]

[1] School of Flexible Electronics (Future Technologies), Nanjing Tech University, Nanjing, China
* Correspondence: iamhbsun@njtech.edu.cn

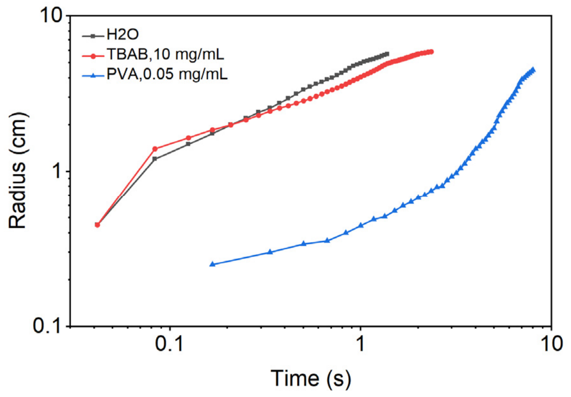

Figure S1. Temporal evolution of the spreading radius of a 20 mg/mL P3HT/chlorobenzene solution on different aqueous subphases. The aqueous subphases include deionized water, a 10 mg/mL TBAB aqueous solution, and a 0.05 mg/mL PVA aqueous solution. The PVA has a weight-average molecular weight (Mw) of 13,000–23,000 g·mol^{-1} and a degree of hydrolysis (DH) of 98%.

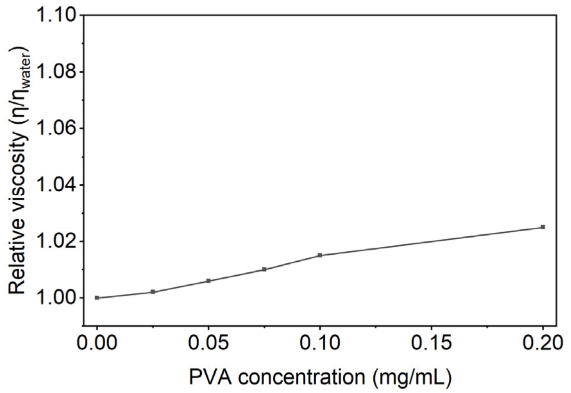

Figure S2. Concentration-dependent relative viscosity of low-molecular-weight PVA aqueous solutions measured using an Ubbelohde viscometer at 25 °C. The PVA has a weight-average molecular weight (Mw) of 13,000–23,000 g·mol^{-1} and a degree of hydrolysis (DH) of 98%. The viscosity values were normalized to that of deionized water.

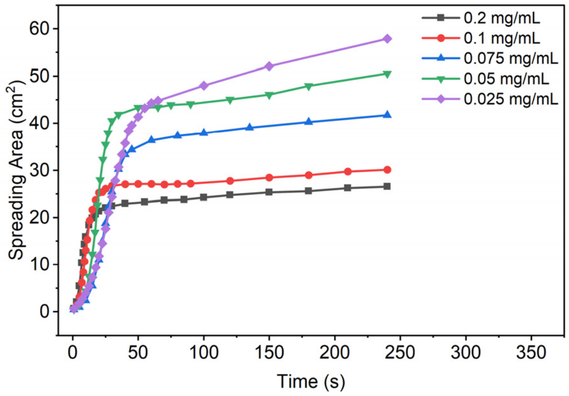

Figure S3. Time-dependent spreading area of a 20 mg/mL P3HT/chlorobenzene solution on PVA aqueous subphases with different concentrations under a chlorobenzene vapor atmosphere. The PVA has a weight-average molecular weight (Mw) of 13,000–23,000 g·mol^{-1} and a degree of hydrolysis (DH) of 98%.

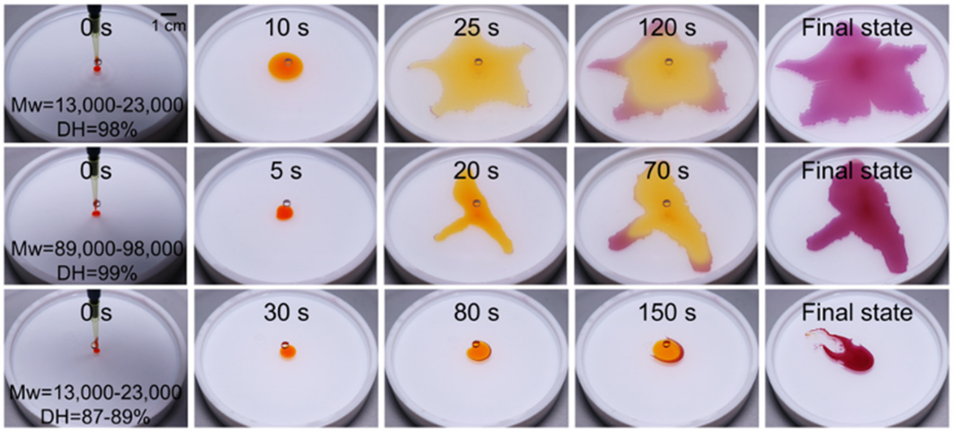

Figure S4. Spreading and film-formation processes of a 20 mg/mL P3HT/chlorobenzene solution on 0.05 mg/mL PVA aqueous subphases with different molecular weights and degrees of hydrolysis under a chlorobenzene vapor atmosphere. The aqueous subphases are, in sequence, a PVA aqueous solution with Mw = 13,000–23,000 g·mol^{-1} and DH = 98%; a PVA aqueous solution with Mw = 89,000–98,000 g·mol^{-1} and DH = 99%; and a PVA aqueous solution with Mw = 13,000–23,000 g·mol^{-1} and DH = 87–89%. The scale bar is 1 cm.

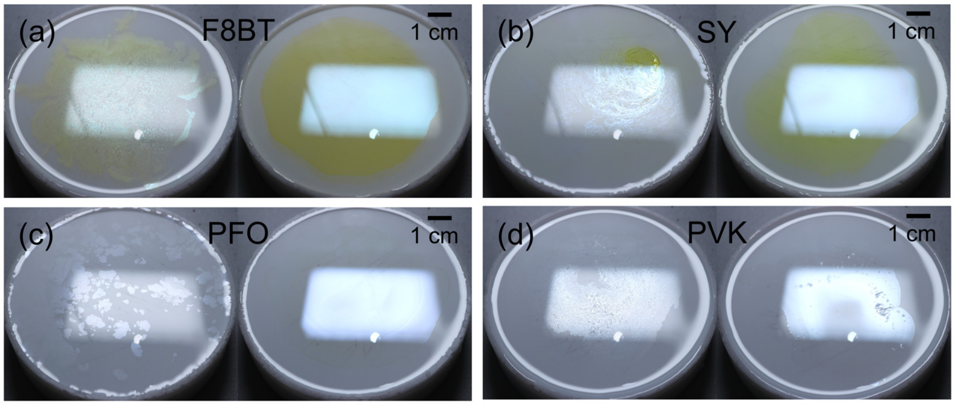

Figure S5. Comparison of spreading and film formation of different polymer/chlorobenzene solutions on deionized water and PVA aqueous solution surfaces. In each panel, the left image shows the film formed on deionized water, and the right image shows the film formed on the PVA aqueous solution. The polymer solutions were: (a) 10 mg/mL F8BT in chlorobenzene; (b) 10 mg/mL SY in chlorobenzene; (c) 20 mg/mL PFO in chlorobenzene; and (d) 20 mg/mL PVK in chlorobenzene. The PVA aqueous solution had a concentration of 0.05 mg/mL, with Mw = 13,000–23,000 g·mol−1 and DH = 98%. Scale bars: 1 cm.

Table S1. Surface tension, interfacial tension, and spreading coefficient of 0.05 mg/mL PVA aqueous solutions with different molecular weights and degrees of hydrolysis.

PVA substrate	Gas condition	Aqueous surface tension (mN/m)	Organic surface tension (mN/m)	Interfacial tension (mN/m)	Spreading coefficient (mN/m)
Mw = 13,000–23,000 g·mol^{-1}, DH = 87-89%	Air	69.03	33	17.07	18.96
	Chlorobenzene	57.40	33	17.07	7.33
Mw = 13,000–23,000 g·mol^{-1}, DH = 98%	Air	64.24	33	21.94	9.30
	Chlorobenzene	62.46	33	21.94	7.52
Mw = 89,000–98,000 g·mol^{-1}, DH = 99%	Air	63.47	33	23.87	6.60
	Chlorobenzene	61.92	33	23.87	5.05

The spreading coefficient was calculated according to $S = \gamma_1 - \gamma_2 - \gamma_{12}$.

https://doi.org/10.3390/xxxxx
